# Multiple Marker Detection in Peripheral Blood for NSCLC Diagnosis

**DOI:** 10.1371/journal.pone.0057401

**Published:** 2013-02-26

**Authors:** Paola Ulivi, Laura Mercatali, Gian-Luca Casoni, Emanuela Scarpi, Lauro Bucchi, Rosella Silvestrini, Stefano Sanna, Marco Monteverde, Dino Amadori, Venerino Poletti, Wainer Zoli

**Affiliations:** 1 Biosciences Laboratory, IRCCS Istituto Scientifico Romagnolo per lo Studio e la Cura dei Tumori, Meldola, Italy; 2 Interventional Pulmonology, Department of Thoracic Diseases, Morgagni-Pierantoni Hospital, Forlì, Italy; 3 Unit of Biostatistics and Clinical Trials, IRCCS Istituto Scientifico Romagnolo per lo Studio e la Cura dei Tumori, Meldola, Italy; 4 Romagna Cancer Registry, IRCCS Istituto Scientifico Romagnolo per lo Studio e la Cura dei Tumori, Meldola, Italy; 5 General Thoracic Surgery, Department of Thoracic Diseases, Morgagni-Pierantoni Hospital, Forlì, Italy; 6 Department of Medical Oncology, IRCCS Istituto Scientifico Romagnolo per lo Studio e la Cura dei Tumori, Meldola, Italy; University of Barcelona, Spain

## Abstract

**Background:**

Non-invasive early detection of lung cancer could reduce the number of patients diagnosed with advanced disease, which is associated with a poor prognosis. We analyzed the diagnostic accuracy of a panel of peripheral blood markers in detecting non small cell lung cancer (NSCLC).

**Methods:**

100 healthy donors and 100 patients with NSCLC were enrolled onto this study. Free circulating DNA, circulating mRNA expression of peptidylarginine deiminase type 4 (PAD4/PADI4), pro-platelet basic protein (PPBP) and haptoglobin were evaluated using a Real-Time PCR-based method.

**Results:**

Free circulating DNA, PADI4, PPBP and haptoglobin levels were significantly higher in NSCLC patients than in healthy donors (p<0.0001, p<0.0001, p = 0.0002 and p = 0.0001, respectively). The fitted logistic regression model demonstrated a significant direct association between marker expression and lung cancer risk. The odds ratios of individual markers were 6.93 (95% CI 4.15–11.58; p<0.0001) for free DNA, 6.99 (95% CI 3.75–13.03; p<0.0001) for PADI4, 2.85 (95% CI 1.71–4.75; p<0.0001) for PPBP and 1.16 (95% CI 1.01–1.33; p = 0.031) for haptoglobin. Free DNA in combination with PPBP and PADI4 gave an area under the ROC curve of 0.93, 95% CI = 0.90–0.97, with sensitivity and specificity over 90%.

**Conclusions:**

Free circulating DNA analysis combined with PPBP and PADI4 expression determination appears to accurately discriminate between healthy donors and NSCLC patients. This non-invasive multimarker approach warrants further research to assess its potential role in the diagnostic or screening workup of subjects with suspected lung cancer.

## Introduction

Lung cancer is the leading cause of cancer death worldwide and the non-small-cell lung cancer subtype (NSCLC) accounts for about 80% of all cases. The 5-year survival rate is only about 16% for patients diagnosed with advanced lung cancer compared to 70–90% when the disease is diagnosed and treated at earlier stages [1], [2]. Early detection could therefore represent a promising strategy to reduce lung cancer mortality. A recent study by the National Lung Screening Trial Research Team reported a 20% reduction in mortality from the use of low-dose spiral computed tomography (CT) in high-risk individuals [3]. However, radiation doses delivered and high costs limit the widespread application of this technique as a screening procedure [4]. Moreover, the high rate of false positives means that a large proportion of individuals undergo unnecessary follow-up and other diagnostic tests, including biopsy, further increasing costs and health risks associated with screening [3]–[5].

The availability of a non-invasive test performed on peripheral blood and capable of discriminating between subjects with and without lung cancer could have two potential uses: first, it could be used as a preliminary screening method to select individuals at high risk of NSCLC who require further investigation with spiral CT, and second, it could help to discriminate between neoplastic and non-neoplastic disease in subjects with suspect nodules detected by CT scans, thereby eliminating the need for serial CTs or invasive biopsy. It has previously been demonstrated that free circulating DNA, alone or in association with various biomarkers, can distinguish between healthy donors and NSCLC patients with 80–90% sensitivity and specificity [6]–[9]. Other potential biomarkers evaluated in serum, plasma or whole peripheral blood have shown different sensitivity and specificity values [10]–[17]. Pro-platelet basic protein (PPBP, also called connective tissue-activating peptide III [CTAP III] or neutrophil activating protein-2 [NAP-2]), a CXC chemokine member involved in angiogenesis, tumorigenesis and metastasis [18], and haptoglobin, an acute-phase plasma glycoprotein that binds to hemoglobin and prevents oxidative stress [19] have both been identified as potential biomarkers to detect preclinical lung cancer [20]. Moreover, increased peptidylarginine deiminase type 4 (PAD4/PADI4) expression, known to be involved in the post-translational conversion of peptidylarginine to citrulline and also in the repression of p53 regulated genes via citrullination of histones at gene promoters [21] has been observed in the blood and tissue of several malignant tumors. In particular, higher levels of PADI4 have been observed in the peripheral blood of NSCLC patients with respect to healthy individuals [22].

The present study aimed, for the first time, to define the diagnostic accuracy of free circulating DNA in combination with mRNA expression levels of PPBP, haptoglobin and PADI4 in the peripheral blood of NSCLC patients.

## Materials and Methods

### Case Series

One hundred patients with histologically or cytologically confirmed NSCLC referred to the Department of Diseases of the Thorax of Morgagni-Pierantoni Hospital in Forlì were enrolled onto the study. The control group consisted of 100 healthy donors enrolled from the Blood Transfusion Unit of the same hospital during the same period. Both healthy donors and patients did not have a previous history of malignant disease. Clinical-pathological characteristics of patients and healthy donors are reported in Table 1.

**Table 1:** Patient and healthy donor characteristics.

	Healthy donors *n*	NSCLC patients *n*
**Overall**	100	100
**Gender**		
M	47	74
F	53	26
**Age (years)**		
≤65	49	18
>65	51	82
**Smoking habits**		
Never	46	11
Former	10	28
Smokers	28	42
Missing	16	19
**Histotype**		
ADC		46
SCC		44
ADC+SCC		1
Other		9
**Stage**		
I		42
II		16
III		16
IV		26

Forty-six tumors were adenocarcinomas (ADC), 44 squamous cell carcinomas (SCC), 9 poorly differentiated carcinomas, and 1 was mixed adenosquamous carcinoma. On the basis of TNM classification 42 tumors were stage I, 16 stage II, 16 stage III and 26 stage IV. Blood samples were taken after obtaining written informed consent from all healthy donors and patients, prior to any anticancer treatment in the latter. The study protocol was reviewed and approved by the ‘*Area Vasta’ Istituto Scientifico Romagnolo per lo Studio e la Cura dei Tumori* (*IRST)* Ethics Committee.

### Sample Collection

Five-milliliter samples of peripheral blood from controls and patients were collected in test tubes without anticoagulant, allowed to clot at room temperature for 30 minutes, and centrifuged at 2,500 rpm for 15 minutes. For RNA extraction, 2.5 ml of peripheral blood were collected in PAX-Gene blood RNA tubes (Qiagen), specifically designed for the collection and stabilization of cellular RNA from whole blood.

Samples were stored at −80°C and, on the basis of the results from preliminary experiments, were processed within a maximum of 3 months to avoid biases caused by prolonged sample storage.

### Extraction and Quantification of Free Circulating DNA

DNA was extracted from 1 ml of serum by QIamp DNA Mini Kit (Qiagen), eluted in a final volume of 50 µl of sterile distilled water and stored at −20°C. To quantify the circulating free DNA, a Real-Time quantitative PCR assay based on SYBR Green I dye chemistry and MyiQ Single Color Real-Time PCR Detection System (BioRad) was used.

DNA quantification was assessed by amplifying the single-copy gene glyceraldehyde-3-phosphate dehydrogenase gene (*GAPDH*), as described previously [8]–[23]. Forward and reverse primer sequences used for amplification of *GAPDH* gene were 5′- ACC CAG AAG ACT GTG GAT GG - 3′ and 5′- TTC AGC TCA GGG ATG ACC TT- 3′, respectively. PCR reaction mix was prepared in a total volume of 25 µl containing 1X SYBR Green Supermix (BioRad), 400 nM of each primer and 5 µl of DNA. PCR conditions were set up as follows: a first denaturation at 95°C for 3 minutes, 40 cycles at 95°C for 15 seconds and then at 55°C for 30 seconds for annealing and extension. The PCR amplified products were analyzed by melting curves whose shape is a function of the GC content, length and sequence of the amplified gene fragment. The absolute concentration of target DNA was calculated on a standard curve using concentrations ranging from 0.01 to 25 ng of DNA from the peripheral blood of a healthy donor.

Each sample was run in triplicate and intra-assay variability was assessed by computing the coefficient of variation (CV) among the three C_t_ values (defined as the fractional cycle number at which the emitted fluorescence exceeds a fixed threshold value above the baseline), which was always <1.5%. Inter-assay variability between two independent experiments in which the procedure was repeated using another sample from the same individual was assessed by the CV and was always <15%. All measurements were made blind.

### RNA Extraction and Marker Amplification

RNA was extracted from the blood of controls and patients by PAX-Gene blood RNA kit (Qiagen), according to the manufacturer’s instructions. The quantity of RNA extracted was assessed by Nanodrop (Celbio) and quality was evaluated by random sampling using the Experion system (Bio-Rad). RNA was treated with DNase I (Qiagen) to eliminate all genomic DNA contaminations and 500 ng of RNA were reverse-transcribed using the iScript cDNA Synthesis Kit (BioRad) in a final volume of 20 µl. The reaction was carried out at 42°C for 30 minutes and was stopped by heating to 85°C for 5 minutes. cDNA was diluted 1∶2 and PCR Real Time was performed using TaqMan Gene Expression Assays for PADI4, PPBP, haptoglobin, and the two housekeeping genes, *HPRT1* and *GAPDH.* TaqMan Universal PCR Master Mix, No AmpErase UNG (Applied Biosystems) was also used for all the reactions. PCR reactions were carried out in triplicate using 7500 PCR Real Time System (Applied Biosystems) under the following conditions: 95°C for 10 minutes, and 40 cycles of 95°C for 15 seconds and 60°C for 1 minute.

### Statistical Analysis

Nonparametric ranking statistics (median test) were used to analyze the relationship between median values of free circulating DNA, PADI4, PPBP and haptoglobin mRNA expression and healthy donor and patient characteristics. Spearman’s correlation coefficient (r_s_) was used to investigate the relationship between the different biomarkers considered as continuous variables. The most efficient cut off values to discriminate between healthy donors and cancer patients were identified using receiver operating characteristic (ROC) curve analysis. The true positive rates (sensitivity) were plotted against the false positive rates (1-specificity) for all classification points. 95% confidence intervals (95% CI) were calculated for sensitivity and specificity values. The chi-square test was performed to evaluate the differences in sensitivity and specificity between the clinical and smoking habit subgroups.

Combined marker analysis was performed using the most discriminating cut off values: 25 ng/ml for DNA, 60 for PPBP, 70 for haptoglobin and 100 for PADI4.

We considered the tests negative when all markers were below their relative cut off value and positive when at least one marker was above its relative cut off value.

The independent diagnostic relevance of markers considered as continuous variables was analyzed by the logistic regression model in which natural logarithmic concentrations of markers were considered as predictor variables, and cancer status (case/control) was considered as a binary outcome variable [24]. The linear predictor or logit resulting from this multivariable model after stepwise procedure was used as a new diagnostic test for which the ROC curve was calculated. A graphical representation of the sensitivity of individual demographic characteristics at a fixed 90% specificity was made (Forest plot).

All p values were based on two-sided testing and statistical analyses were carried out using SAS Statistical software version 9.1 (SAS Institute).

## Results

### Marker Analysis

All four markers investigated were significantly higher in NSCLC patients than in healthy controls. Median values of free circulating DNA were 47.2 ng/ml (range, 0.7–251) for patients and 9.2 ng/ml (range, 2.2–184) for healthy donors (p<0.0001); PADI4 relative expression was 69.9 (range, 19–403.9) and 36.5 (range, 5.9–143.9) (p<0.0001), PPBP relative expression was 34.2 (range, 8.8–212.7) and 23.5 (range, 8.6–58.7) (p = 0.002), and haptoglobin relative expression was 30 (range, 1–487) and 17.4 (range, 1–62) (p<0.0001), respectively. None of the markers were correlated with gender, age or smoking habits in either group or with tumor histotype or disease stage in NSCLC patients.

**Figure 1:**
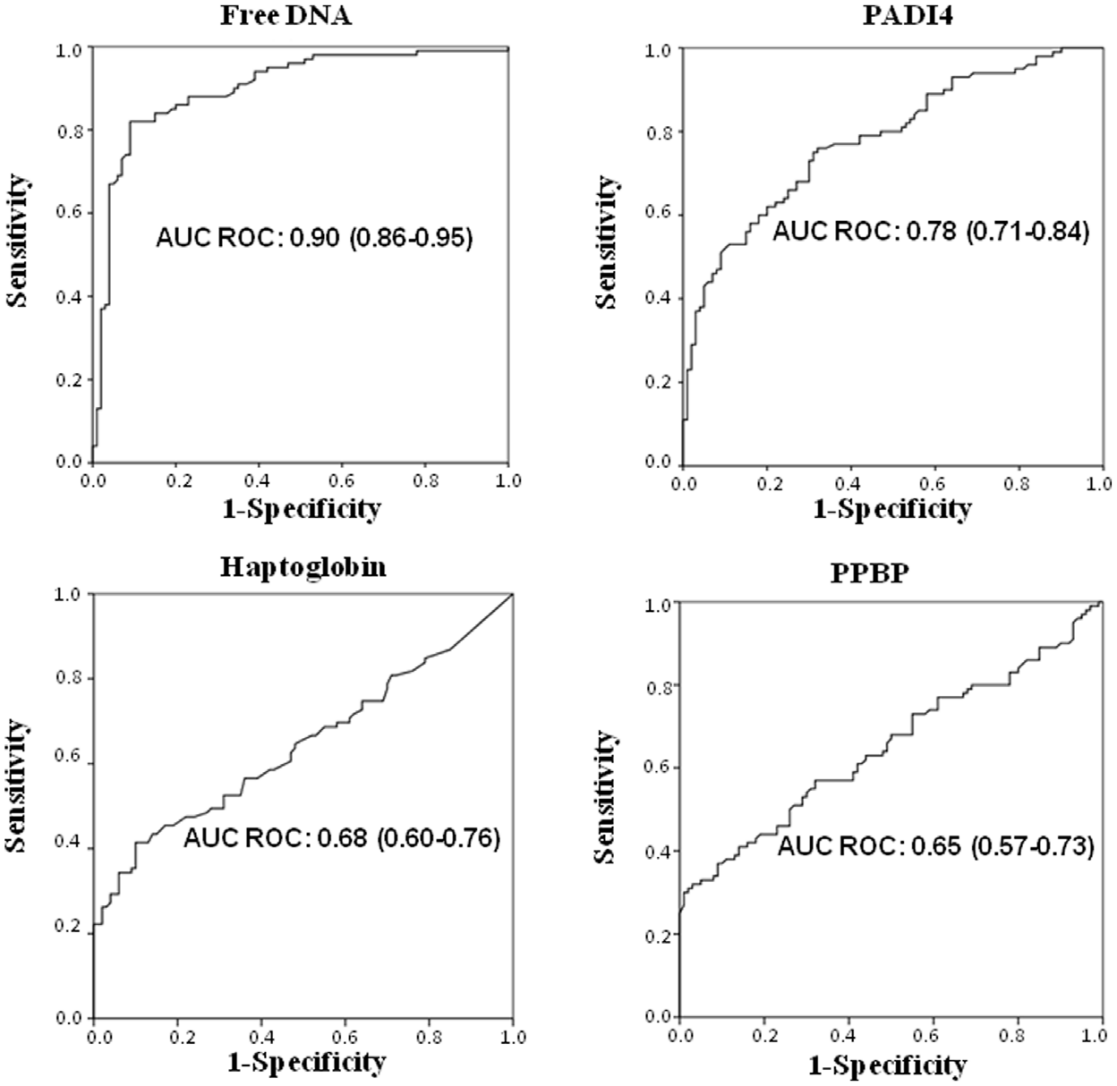
ROC curves of free DNA, PADI4, PPBP and haptoglobin.

The relation between marker expression and risk of lung cancer, analyzed by the fitted logistic regression model, showed odds ratios (Ors) of 6.93 (95% CI 4.15–11.58; p<0.0001) for free DNA, 6.99 (95% CI 3.75–13.03; p<0.0001) for PADI4, 2.85 (95% CI 1.71–4.75; p<0.0001) for PPBP and 1.16 (95% CI 1.01–1.33; p = 0.031) for haptoglobin. Moreover, the diagnostic accuracy of different markers showed an area under the ROC curve (AUC) of 0.90 (0.86–0.95) for free DNA, 0.78 (0.71–0.84) for PADI4, 0.68 (0.60–0.76) for haptoglobin and 0.65 (0.57–0.73) for PPBP (Figure 1).

The best sensitivity and specificity values were observed for free circulating DNA, which showed 85% and 82% sensitivity and 80% and 91% specificity at 20 ng/ml and 25 ng/ml cut off values, respectively. PADI4, PPBP and haptoglobin were characterized by generally high specificity (90% to 100%) but very low sensitivity (20% to 40%) for PADI4 and PPBP, and even lower (around 20%–30%) for haptoglobin. Specificity >90% was observed for PADI4 cut offs >70 but was associated with a sensitivity of around 50%. Cut off values >50 for PPBP and >40 for haptoglobin showed 91% and 100% specificity, respectively, but only about 30% sensitivity (Table 2).

**Table 2:** Sensitivity and specificity of the different markers.

Free DNA cut off values (ng/ml)	Sensitivity (%)	*95% CI*	Specificity (%)	*95% CI*
** 20**	**85**	*77–91*	**80**	*72–87*
** 25**	**82**	*74–89*	**91**	*84–96*
** 30**	**74**	*65–82*	**92**	*86–96*
**PADI4 cut off values (relative expression)**				
** 80**	**43**	*34–53*	**94**	*88–98*
** 90**	**33**	*24–42*	**97**	*92–99*
** 100**	**23**	*15–32*	**98**	*94–100*
**PPBP cut off values (relative expression)**				
** 40**	**42**	*33–52*	**82**	*74–89*
** 50**	**34**	*25–44*	**91**	*25–44*
** 60**	**25**	*17–34*	**100**	*–*
**Haptoglobin cut off values (relative expression)**				
** 50**	**27**	*19–36*	**96**	*91–99*
** 60**	**23**	*15–32*	**98**	*94–100*
** 70**	**21**	*14–30*	**100**	*–*

### Combined Score

**Figure 2:**
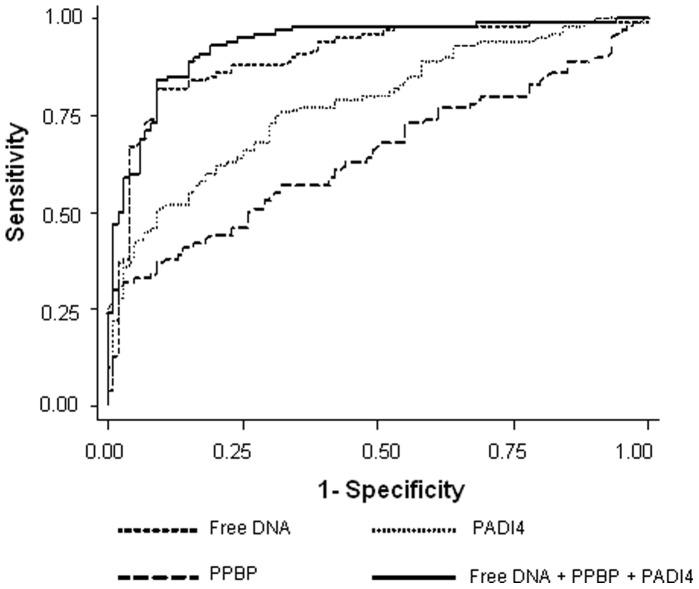
ROC curve analysis of free circulating DNA, PADI4 and PPBP as single markers or in combination.

We first analyzed the relationship between the different markers; free DNA was not correlated with any other marker, and PPBP was not correlated with haptoglobin or PADI4. The only significant association observed was that between haptoglobin and PADI4 (r_s_ = 0.318, p = 0.003). Therefore, on the basis of these findings and in an attempt to improve its diagnostic accuracy, free DNA was analyzed in combination with the other markers. When the markers were used as continuous variables, transformed into natural logarithmic values and analyzed in a multiple logistic regression model, adjusted for age, gender and smoking habits, all but haptoglobin provided independent diagnostic information (data not shown). When a combination of DNA, PPBP and PADI4 was considered, a unit increase in log DNA, log PPBP or log PADI4 was associated with a six-, five- and two-fold increase in cancer risk, respectively (Table 3). The combination of PADI4 or PPBP with free DNA did not increase the diagnostic accuracy of free DNA alone (data not shown). Conversely, a significant increase in both sensitivity and specificity was obtained when all three markers were considered together (AUC 0.93, 95% CI = 0.90–0.97; p = 0.01) (Figure 2). Although the increase in diagnostic accuracy was very modest, it must be underlined that 7 of the 10 patients identified only with the combination strategy had early stage disease (2 stage I and 5 stage II). In particular, we chose cut off values characterized by almost absolute specificity for PADI4 and PPBP, albeit coupled with low sensitivity, in an attempt to increase free DNA sensitivity without decreasing its specificity. Specifically, the triple marker combination showed a 92% sensitivity and 89% specificity in the overall series and a higher diagnostic accuracy was consistently observed in all the clinical-pathological subgroups (Table 4).

**Table 3:** Combined analysis of markers considered as continuous variables in a multiple logistic regression model adjusted for age, gender and smoking habits.

Variable	Coefficient (s.e.)	Wald test	Odds Ratio(95%CI)	p
Constant	−15.557 (2.464)	−6.31	–	–
ln DNA	1.913 (0.297)	6.43	6.771 (3.780–12.130)	<0.0001
ln PADI4	1.632 (0.419)	3.89	5.115 (2.248–11.639)	<0.0001
ln PPBP	1.016 (0.373)	2.72	2.763(1.329–5.744)	0.007

s.e., standard error.

**Table 4:** Diagnostic accuracy of DNA combined with PPBP and PADI4 in relation to clinical (all cases) and pathological (patients) characteristics.

DNA 25 ng/ml+PPBP 60+ PADI4 100				
	Sensitivity% (*n)*	95% CI	Specificity% (*n)*	95% CI
**All cases**	**92** (92/100)	86–96	**89** (89/100)	82–94
**Gender**				
M	**95** (70/74)	88–98	**85** (40/47)	73–93
F	**96** (25/26)	84–100	**93** (49/53)	83–98
**Age (years)**				
≤65	**100** (18/18)	–	**84** (41/49)	72–92
>65	**90** (74/82)	83–95	**94** (48/51)	86–99
**Smoking habits**				
Never	**91** (10/11)	66–99	**87** (40/46)	75–95
Former	**89** (25/28)	75–97	**100** (10/10)	–
Smokers	**95** (40/42)	86–99	**89** (25/28)	75–97
**Histotype**				
ADC	**91** (42/46)	81–97		
SCC	**86** (38/44)	74–94		
**Stage**				
I	**93** (39/42)	83–98		
II	**100** (16/16)	–		
III	**94** (15/16)	75–100		
IV	**85** (22/26)	68–95		

**Figure 3:**
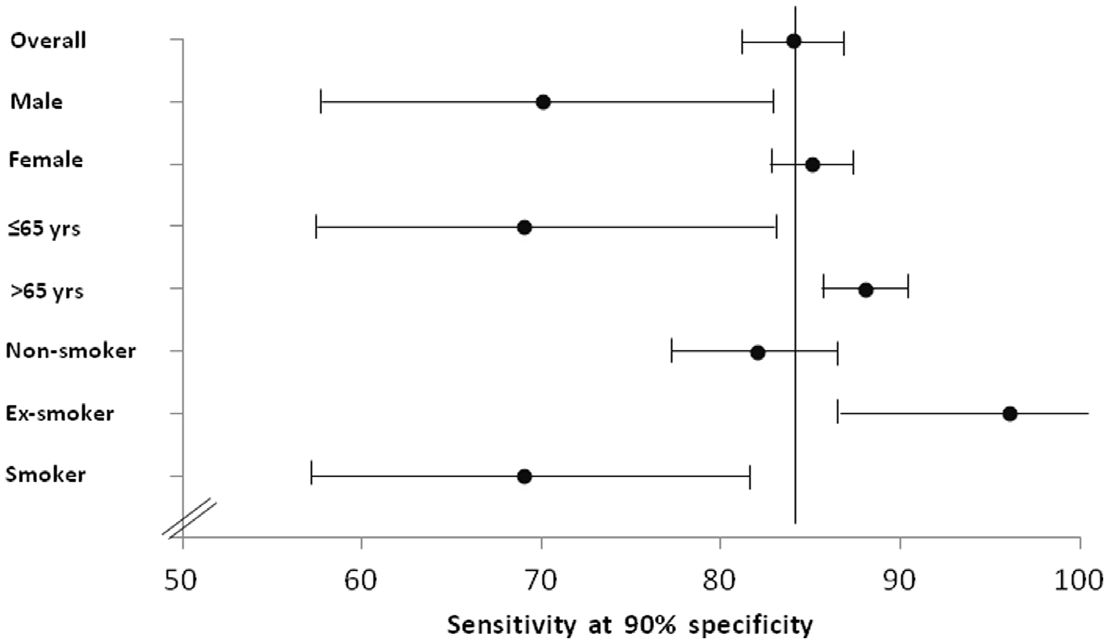
Forest Plot showing biomarker sensitivity at a fixed 90% specificity in relation to patient and healthy donor clinical characteristics.

Finally, Forest plot analysis, showing the sensitivity at a fixed 90% specificity by gender, age and smoking status of patients and healthy donors, did not demonstrate any significant differences in sensitivity on the basis of demographic characteristics of patients (Figure 3) (p>0.10).

## Discussion

Technical advancements have made spiral CT scans more accurate than any other diagnostic approach in detecting lung tumors at a resectable stage, inducing a 20% of mortality reduction [3]. However, the high sensitivity of spiral-CTs is coupled with low specificity, which often results in benign lesions, appearing suspect and requiring close follow up to evaluate any changes [3], [25], [26]. Other instrumental diagnostic approaches, PET imaging, fine needle aspiration or bronchoscopy, can be used for the differential diagnosis of suspect nodules, but the cost and invasiveness of these approaches limit their widespread use in screening programs.

Whilst an inexpensive and minimally invasive test would be ideal to use in conjunction with spiral-CT scans in screening projects or in all cases of dubious diagnosis, no such test exists with FDA approval. A number of studies have demonstrated that free circulating DNA levels are higher in NSCLC patients than in healthy donors [6]–[9], [23]. In Zhang and coworkers’ metanalysis of ten studies focusing on the diagnostic accuracy of free DNA, mean sensitivity and specificity values of 80% and 77%, respectively, were reported, with an overall ROC curve value of 0.89. Similar results were obtained using different methodologies, such as PCR, fluorimetric assay or ELISA [9]. Moreover, in the different studies, no correlation was found between free DNA level and tumor stage, reinforcing the former’s role as a diagnostic marker [6]–[8]. Although baseline assessment of plasma DNA level does not improve the accuracy of lung cancer screening by spiral CT in heavy smokers [27] this marker is a potentially important tool in that it is capable of distinguishing between cancer and non cancer patients.

In the present study, we aimed to increase the diagnostic accuracy of free DNA by using it in combination with other circulating biomarkers. Haptoglobin and PPBP have previously been shown to be good diagnostic biological indicators of NSCLC, their diagnostic accuracy increasing when they are evaluated in combination with clinical factors such as FEV_1_, sex and age [20]. PPBP belongs to the subfamily of CXC chemokines, which are potent promoters of angiogenesis, tumorigenesis and metastases [18]. Moreover, it has been demonstrated that PADI4 expression is increased in the peripheral blood of patients with lung, breast, colorectal and bladder cancer, highlighting its potential involvement in the process of tumorigenesis [22]. Although our main aim was to define molecular markers that can be used for the early detection of NSCLC, we also analyzed patients at any stage of disease in order to evaluate the trend of the different markers in different stages. The independence of each marker from the stage of disease reinforced their potential usefulness for the non invasive early detection of cancer.

Our comparative analysis of single markers showed the diagnostic superiority of free DNA in terms of sensitivity and specificity. Furthermore, the combined analysis of DNA with the other markers significantly improved diagnostic accuracy to over 90%. The combination of DNA, PPBP and PADI4 was the most effective, resulting in a significant increase in diagnostic accuracy with respect to that obtained using free DNA alone, with sensitivity and specificity ranging from 93% to 100% in the overall series and in all clinical-pathological subgroups. Specifically, the addition of PPBP or PADI4 to free DNA did not substantially increase diagnostic accuracy with respect to free DNA alone, whereas the three markers analyzed together significantly improved free DNA diagnostic potential. This is probably due to the fact that the different markers, unrelated to each other, made independent positive contributions.

The 82% sensitivity and 91% specificity observed for free DNA alone are similar to results reported in a number of studies [7], [8], [28], [29] but higher than those described in others [30]–[32]. This discordance may be ascribable to differences in biological material used (plasma or serum), in control populations, or in the methodologies applied. The diagnostic accuracy of free DNA in combination with PPBP and PADI4 was higher than that of DNA alone and enabled us to identify 10% more NSCLC patients, the majority of whom had early stage disease.

The sensitivity and specificity percentages obtained using the combination analysis are similar to those previously observed by our group by combining DNA with COX-2 expression [8]. This indicates that free DNA is the most useful diagnostic marker for lung cancer and that the combination of DNA with either PPBP and PADI4 or COX2 could improve diagnostic accuracy, especially of early stage tumors.

The majority of published studies use healthy donors as the control group, only a few [28], [33] considering individuals with non-neoplastic lung diseases. In the latter studies, a lower diagnostic accuracy of free DNA was observed because DNA levels were higher in patients with non malignant lung diseases than in healthy donors. Furthermore, we previously showed that free DNA levels are also very high in patients with idiophatic pulmonary fibrosis (IPF) [34], suggesting that this marker cannot be used for the differential diagnosis between IPF and NSCLC. The role of our three-marker combination in patients with non malignant lung disease requires further evaluation.

In conclusion, our results open up interesting prospects for these markers as an inexpensive and minimally invasive method for the early detection of lung cancer or for the evaluation of cases of dubious diagnosis or suspect nodules detected by spiral CT.
